# Establishment of a fit prediction model of N95 respirator based on facial images

**DOI:** 10.1265/ehpm.25-00258

**Published:** 2025-12-15

**Authors:** Guifang Wang, Changwei Luo, Can Cui, Shengjin Wang, Jing Huang

**Affiliations:** 1 Department of Infection Control, Beijing You’an Hospital, Capital Medical University, No. 8, Xitoutiao, You’anmen Wai, Fengtai District, Beijing, China, 100069; 2 Department of Electronic Engineering, Tsinghua University, No. 30, Shuangqing Road, Qinghuayuan Street, Haidian District, Beijing, China, 100084; 3 Academy of Military Sciences, No. 1, Xianghongqi Dongmenwai, Haidian District, Beijing, China, 100091; 4 Department of Infection Control, Peking Union Medical College Hospital, Chinese Academy of Medical Sciences, Peking Union Medical College Hospital, No. 1 Shuaifuyuan Wangfujing Dongcheng District, Beijing, China, 100730

**Keywords:** N95 respirator, Facial dimensions, Infection control, Respiratory protection

## Abstract

**Background:**

The protective effectiveness of an N95 respirator depends on the filtration efficiency of the material from which the N95 respirator is made of, as well as the wearers’ facial fit. The facial fit of an N95 respirator mainly depends on the degree of matching between the wearers’ facial dimension characteristics and the N95 respirator. Quantitative fit testing objectively evaluates the fit of N95 respirators; however, it is not easy to promote because of the limitations of testing conditions. The aim of this study is to establish a fit prediction model of N95 respirator based on facial images.

**Methods:**

Facial images and fit factor (FF) value of 5 N95 respirators were gathered from 299 medical staffs in 10 hospitals in Beijing. Face geometry measurement was based on 3D face modelling, and the American TSI-8038 Porta Count Pro+ was used to conduct quantitative fit test. Multiple linear regression analysis was employed to identify facial dimensional features that significantly influenced the fit of N95 respirators. Through matching training of facial image and FF values, a fit prediction model has been established, enabling rapid recommendation of N95 respirators meeting the fit standard via facial image recognition.

**Results:**

A fit prediction model for N95 respirators based on facial images has been developed, which enables the rapid recommendation of N95 respirators with acceptable FF value for healthcare personnel. The model demonstrated an accuracy of 55.93%, a precision of 98.43%, a recall of 51.65%, and an F1 score of 0.68.

**Conclusions:**

It is feasible to utilize computer-based facial recognition technology to rapidly recommend N95 respirators for medical personnel. Given the high level of accuracy achieved, the model demonstrates significant potential for practical application.

The N95 respirator is the most important protective device for preventing respiratory transmitted diseases. Limited studies have shown that less than 50% of N95 respirator wearers achieve the desired protective efficacy. The protective efficacy of N95 respirators depends on the filtration efficiency of the protective material and the suitability of the protective device to the wearer’s face. Quantitative suitability testing can objectively evaluate the suitability, but it is difficult to promote due to the limitations of testing conditions and other reasons [1–6]. Therefore, how to quickly select an N95 respirator with the suitability standard has become an urgent problem to be solved for the prevention and control of new infectious diseases. The suitability mainly depends on the size of the wearer’s head and face and the matching degree of the mask. Selecting an appropriate N95 respirator and wearing it correctly are two key steps to ensuring protection effectiveness. Kim, D. et al. developed an artificial intelligence system that uses thermal imaging to quickly detect whether medical staff are wearing masks correctly, while this study aims to achieve rapid selection of appropriate N95 respirator [7]. Based on computer vision technology, this study tested the feasibility of the wearer’s face size, identified the facial images of medical personnel, quantitatively tested the suitability of different medical protective masks, and found the types and models of N95 respirators for people with different facial dimensions through the measurement results. A fit prediction model based on the wearer’s facial image was established to quickly recommend the N95 respirator with the standard of fitness, and improve the ability of prevention and control of infectious diseases [8–10].

## Methods

### Research design

**Fig. 1:**
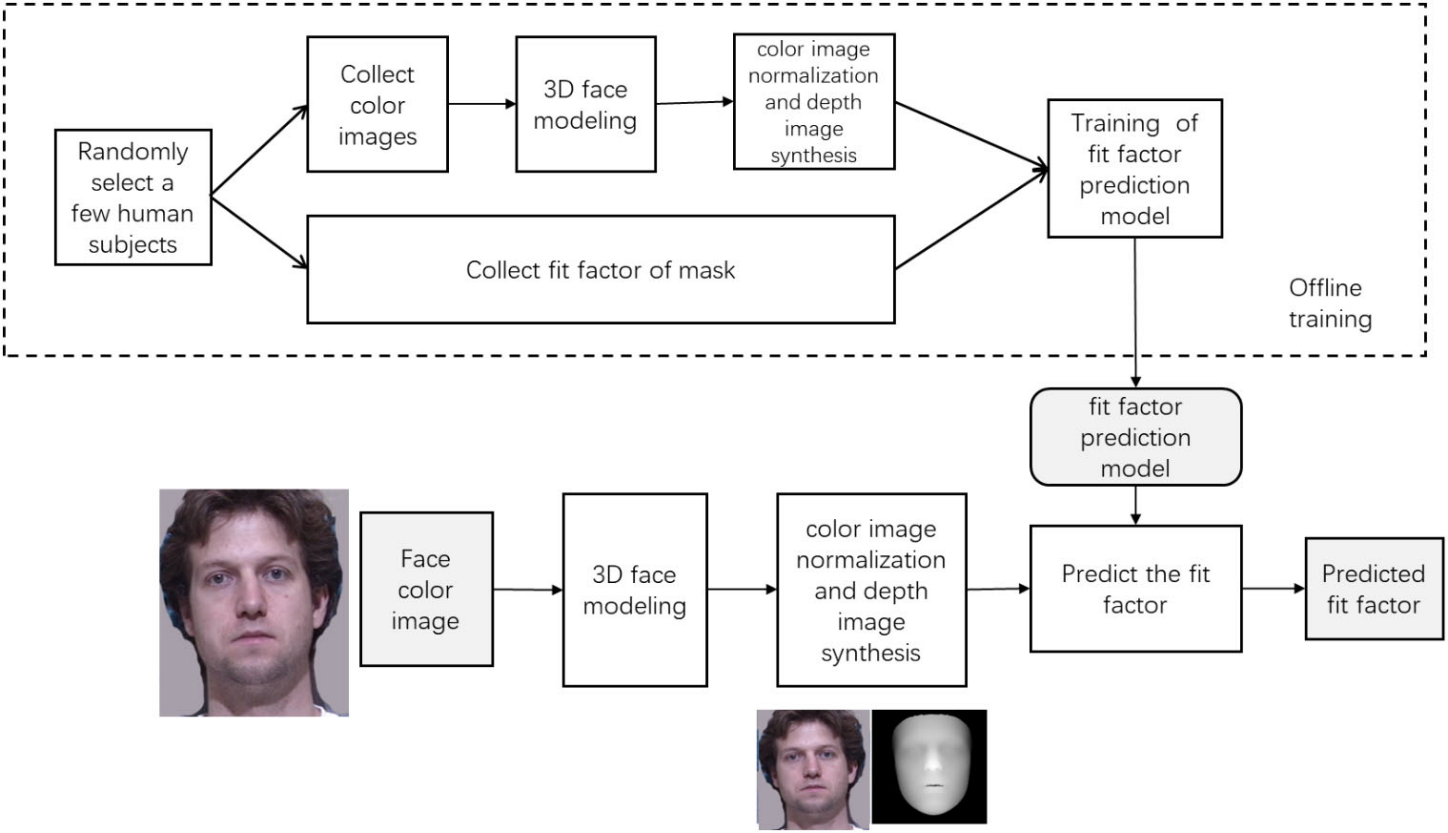
The technical roadmap

This study is a quantitative, cross-sectional observational design and it conducted facial image acquisition and facial 3D feature measurements for medical staff and quantitatively tested the facial fit of five different N95 respirators. By matching and training the facial fit test results with the wearers’ facial images, a fit prediction model was established to rapidly select the N95 respirators that satisfied the fit standard through facial image recognition technology. Figure 1 shows the design ideas.

### Participant selection

A total of 299 healthcare professionals participated in the development of the model. including wearing five different N95 respirators for quantitative fit tests and receiving facial image acquisition. The inclusion criteria were as follows: normal activity of all body parts, normal cardiopulmonary function, normal language function, and tolerance to wearing various types of N95 respirators for a long time (more than 4 hours; men needed to shave off their beards. The exclusion criteria were as follows: limited head/neck or body movement, limited speech function, limited facial muscle movement, inability to sit or stand up, inability to tolerate wearing the N95 respirator for a prolonged period (more than 4 hours), and significant facial defects that prevented respirators from fitting.

### N95 respirator selection

Five types of N95 respirators that met the “Technical requirements for protective face mask for medical use” in GB 19083-2010 and sold well in the Chinese domestic market were selected for use in this project, including 3M 9132 (folding, Shanghai 3M Company), SW D918 (folding, Shandong Sinuo Medical Instrument Co., Ltd), NTPN95N (flat, Naton Medical Group), WN-N95 fold style (folding, Winner Medical), and WN-N95 cup style (cup, Winner Medical). Products to be included in the study were required to be of good quality, in good packaging and within their expiry date. Products with damaged outer packaging or past their shelf life were not included in the study.

### Facial image acquisition

**Fig. 2:**
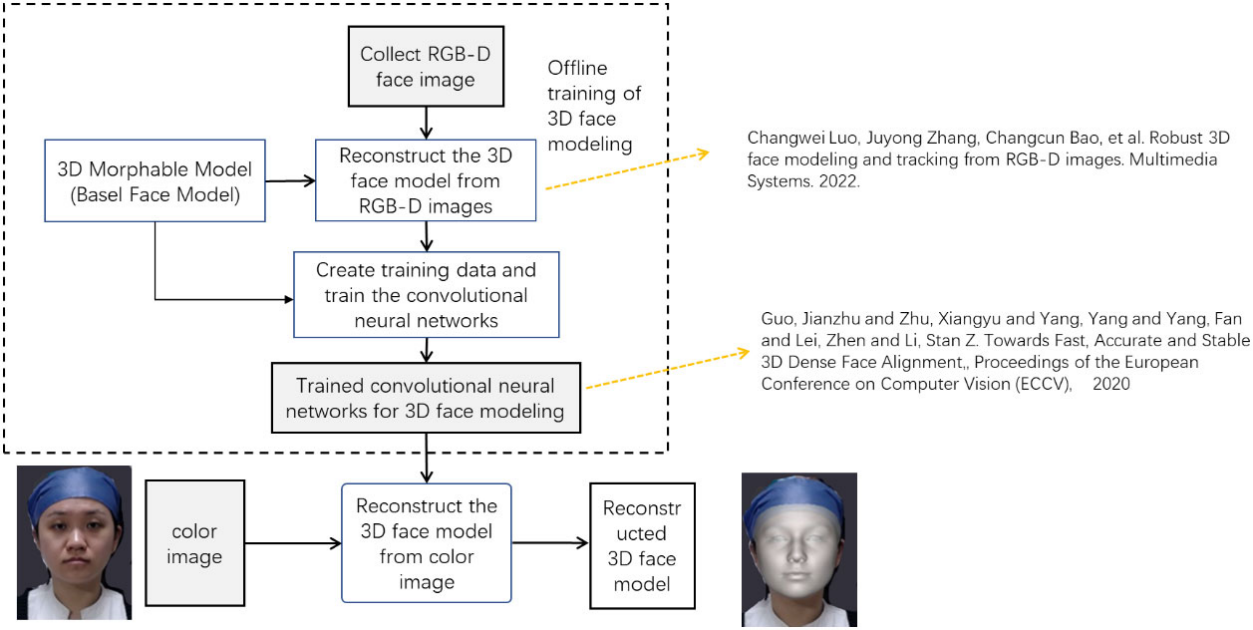
Flowchart of facial image extraction

**Fig. 3:**
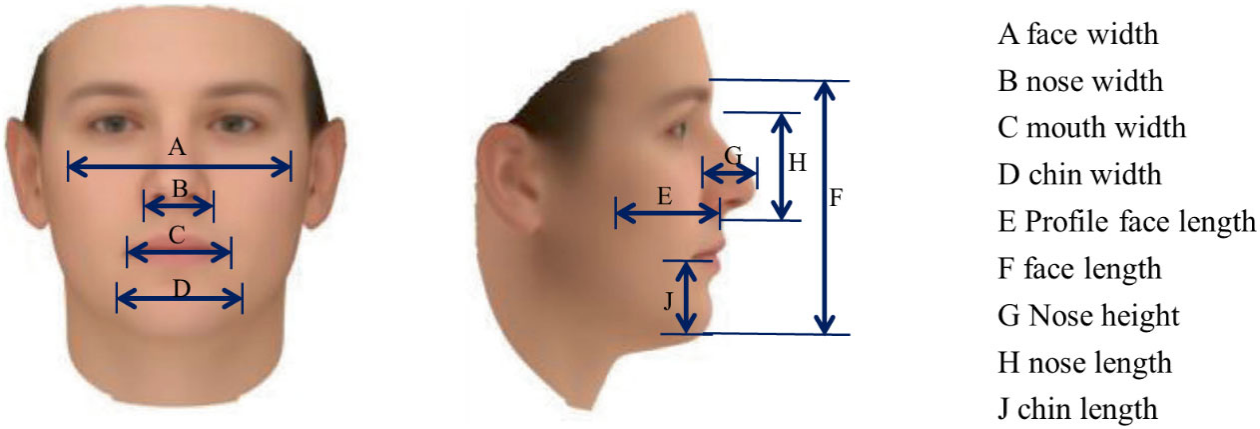
Facial dimension measurement schematic

In the process of extracting facial dimensions, an Intel RealSense D435 camera (Intel Corporation, Santa Clara, California, USA.) was employed to capture colour image of the subject, the method of the following paper is used to reconstruct a detailed 3D face model of the subject. Then, by defining features such as head length, head width, and morphological surface length based on coordinates derived from grid points in this reconstructed 3D face model, corresponding facial dimensions are calculated. This approach enables assessment of similarity between two faces through integration of both 3D facial measurement data and face similarity metrics. Figure 2 shows the facial image extraction process and Fig. 3 shows the measurement criteria for the facial dimensions [11].

### Quantitative fit test

The quantitative fit test was performed on the test participants using TSI-8038 Porta Count Pro+ (TSI, Shoreview, MN, USA). In the fit test, the test participants completed the following six specified actions in sequence: (1) normal breathing; (2) deep breathing; (3) turning the head around and around; (4) nodding up and down; (5) speaking loudly; and (6) breathing normally. The instrument automatically calculated the fit factor value of each action and the overall FF. The FF value ranges from 0 to 200 in the N95 test mode. The provisions of OSHA 29CFR 1910.134 Appendix A Part I.C.3 (September 26, 2019) indicate that the FF value of half-surface respirator is ≥100, which passes the test, whereas their FF value is <100, which fails the test [12].

### Statistical analysis

Independent-sample t test was used for comparisons of continuous data. Classified data were compared between groups using χ2 test. The effect of facial dimensions on the total FF value of N95 respirators was analysed using multiple linear regression. The test level of selected independent variables (α_in_) was 0.1, and the test level of excluded independent variables (α_out_) was 0.15. The regression model was considered statistically significant at *P* < 0.05. Data analysis was performed using SAS statistical analysis software version 9.1 (SAS Inc., Cary, NC, USA).

## Results

### Demographic information of participants

A total of 299 healthcare workers participated in the development of the model which from Beijing, China, with 140 male and 159 female aged 18–60 years. The demographic information of the 299 participants was showed in Table 1.

**Table 1:** Demographic information of the participants

**Characteristic**	**Male**	**Female**	**Total**
Sex	140	159	299
Nation			
Han	130	145	275
Hui	4	5	9
Man	3	8	11
Others	3	1	4
Hometown			
Beijing	56	74	130
Hebei	22	23	45
Shandong	8	12	20
Henan	8	11	19
Shanxi	6	7	13
Others	40	32	72
Professional group			
Doctor	69	35	104
Nurse	17	93	110
Administrative staff	18	18	36
logistical personnel	17	2	19
Others	19	11	30
Age group			
18–25	22	28	50
26–35	37	64	101
36–45	50	33	83
46–60	31	34	65

### Facial dimensions of the participants

Facial dimensions of the 299 participants, including face length, face width, chin length, chin width, nose length, nose width, nose height, profile face width, and mouth width, were measured using computer vision technology. The results of the independent-sample *t* test indicated that chin width and nose height differed between men and women, with men having a wider chin (*P* = 0.032) and higher nose (*P* < 0.0001). However, the remaining facial dimensions were not significantly different. Table 2 presents the specific data on facial dimensions of different sexes and the results of intersex comparison.

**Table 2:** Facial dimensions of 299 participants

**Facial dimensions**	**Male (mean ± SD)**	**Female (mean ± SD)**	**t value**	**P value**
Face length	11.36 ± 0.33	11.29 ± 0.32	−1.90	0.058
Face width	9.79 ± 0.07	9.78 ± 0.06	−1.62	0.107
Chin length	4.11 ± 0.08	4.09 ± 0.09	−2.16	0.032*
Chin width	7.45 ± 0.13	7.43 ± 0.14	−1.38	0.168
Nose length	4.63 ± 0.08	4.62 ± 0.08	−1.58	0.114
Nose width	3.32 ± 0.06	3.31 ± 0.07	−1.55	0.122
Nose height	2.04 ± 0.05	2.01 ± 0.04	−4.85	<0.0001*
Profile face width	10.68 ± 0.14	10.67 ± 0.15	−0.56	0.574
Mouth width	5.66 ± 0.25	5.61 ± 0.25	−1.68	0.094

Note: * indicates a statistically significant difference.

### Fit test results

χ2 test results indicated that the passing rates of the five respirators were not all the same (*P* < 0.0001). Pairwise comparison showed that the passing rates of 3M 9132 and SW D918 were significantly different from those of the other three N95 respirators. No statistically significant differences in the passing rates of NTPN95N, WN-N95 fold style, and WN-N95 cup style respirators were observed. Passing rate of three N95 respirators differed between men and women, with the passing rate of the fit test being higher in men than in women (3M 9132: male, 35.71; female, 18.24; *P* = 0.0006; SW D918: male, 87.14; female, 77.99; *P* = 0.037; and NTPN95N: male, 62.86; *P* = 0.037; female, 49.69; *P* = 0.022). There were no differences in the passing rates of the other two types of respiratory protective devices (WN-N95 fold-style and WN-N95 cup style respirators) between men and women. Table 3 shows the passing rate of the fit test for different respirators.

**Table 3:** Fit test results of the five N95 respirators

**N95 respirators**	**Passed/Failed** **(pass rate, %)**	**χ2**	***P* value**	**Male**	**Female**	**χ^2^**	***P* value**

				**Passed/Failed** **(pass rate, %)**	**Passed/Failed** **(pass rate, %)**		
3M 9132	79/220 (73.58)	191.34	<0.0001*	50/90 (35.71)	29/130 (18.24)	11.70	0.0006*
SW D918	246/53 (82.27)			122/18 (87.14)	124/35 (77.99)	3.28	0.037*
NTPN95N	167/132 (55.85)			88/52 (62.86)	79/80 (49.69)	5.24	0.022*
WN-N95 Folding	150/149 (50.17)			65/75 (46.43)	85/74 (53.46)	1.47	0.225
WN-N95 Cup	171/128 (57.19)			77/63 (55.00)	94/65 (59.12)	0.52	0.473

Note: * indicates a statistically significant difference.

Pairwise comparison results showed that the passing rates of 3M 9132 and SW D918 were significantly different from those of the other three N95 respirators. No statistically significant differences in the passing rates of NTPN95N, WN-N95 Folding, and WN-N95 Cup were observed.

### Analysis of the relationship between the total FF value of N95 respirators and the facial dimensions of participants

First, differences in facial dimensions were compared between the groups that passed and failed the test. The analysis method used was a two-independent-sample *t* test. The results indicated that among medical workers wearing SW D918 for the fit test, the face length difference between the passed and failed groups was statistically significant; however, the face length of the passing group was longer (*P* = 0.043). With respect to NTPN95N, the chin length was significantly different between the passed and failed groups (*P* = 0.0005). Medical workers wore the 3M 9132 and WN-N95 fold style respirators for the fit test; the nose length in passed group was significantly greater than that in failed group (3M 9132, *P* = 0.002; WN-N95 fold style, *P* = 0.008). As for NTPN95N, the chin length of the pass group was slightly longer than that of the failed group (*P* = 0.0005), and the chin length was slightly wider than that of the failed group (*P* = 0.049). Table 4 shows the results of the pair-to-pair comparison of facial dimension data between the group that passed the test and failed the test while wearing the five types of N95 respirators.

**Table 4:** Comparison of facial dimensions between passed group and failed group

**N95 respirators**	**Facial dimension**	**Passed** **(mean (SD))**	**Failed** **(mean (SD))**	**t value**	***P* value**
3M9132	face length	11.34 (0.35)	11.31 (0.32)	−0.73	0.466
	face width	9.79 (0.07)	9.78 (0.06)	−0.83	0.408
	chin length	4.10 (0.10)	4.09 (0.09)	−0.68	0.499
	chin width	7.43 (0.14)	7.44 (0.13)	0.56	0.574
	nose length	4.65 (0.08)	4.62 (0.08)	−3.20	0.002*
	nose width	3.32 (0.06)	3.32 (0.06)	−0.46	0.648
	nose height	2.04 (0.05)	2.02 (0.05)	−1.99	0.047*
	profile face length	10.68 (0.16)	10.67 (0.15)	−0.82	0.414
	mouth width	5.61 (0.26)	5.64 (0.25)	0.86	0.390

SW D918	face length	11.34 (0.33)	11.24 (0.27)	−2.03	0.043*
	face width	9.78 (0.06)	9.78 (0.06)	−0.86	0.389
	chin length	4.10 (0.09)	4.10 (0.09)	−0.04	0.969
	chin width	7.44 (0.13)	7.43 (0.14)	−0.64	0.523
	nose length	4.63 (0.08)	4.62 (0.08)	−1.00	0.316
	nose width	3.32 (0.06)	3.31 (0.07)	−1.46	0.146
	nose height	2.03 (0.05)	2.02 (0.05)	−1.94	0.054
	profile face length	10.67 (0.15)	10.65 (0.15)	−0.96	0.337
	mouth width	5.63 (0.25)	5.64 (0.25)	0.03	0.973

NTPN95N	face length	11.31 (0.32)	11.33 (0.33)	0.28	0.782
	face width	9.78 (0.06)	9.78 (0.07)	0.44	0.660
	chin length	4.11 (0.09)	4.08 (0.09)	−3.50	0.0005*
	chin width	7.44 (0.13)	7.43 (0.13)	−0.81	0.416
	nose length	4.63 (0.08)	4.62 (0.08)	−0.74	0.457
	nose width	3.32 (0.07)	3.31 (0.06)	−1.98	0.049*
	nose height	2.03 (0.05)	2.02 (0.05)	−1.21	0.229
	profile face length	10.68 (0.15)	10.66 (0.15)	−1.59	0.113
	mouth width	5.63 (0.25)	5.64 (0.25)	0.47	0.641

WN-95 Folding	face length	11.31 (0.31)	11.33 (0.34)	0.47	0.641
	face width	9.79 (0.06)	9.78 (0.07)	−1.39	0.166
	chin length	4.10 (0.10)	4.09 (0.08)	−0.64	0.520
	chin width	7.44 (0.14)	7.44 (0.13)	0.37	0.713
	nose length	4.64 (0.08)	4.61 (0.08)	−2.69	0.008*
	nose width	3.31 (0.07)	3.32 (0.06)	1.09	0.278
	nose height	2.03 (0.05)	2.02 (0.05)	−1.07	0.284
	profile face length	10.67 (0.15)	10.67 (0.15)	0.07	0.947
	mouth width	5.62 (0.26)	5.65 (0.25)	1.16	0.247

WN-95 Cup	face length	11.31 (0.31)	11.34 (0.34)	0.85	0.396
	face width	9.79 (0.06)	9.78 (0.07)	−0.98	0.328
	chin length	4.09 (0.09)	4.10 (0.09)	0.43	0.671
	chin width	7.43 (0.13)	7.45 (0.13)	1.36	0.174
	nose length	4.63 (0.08)	4.62 (0.08)	−1.78	0.077
	nose width	3.32 (0.06)	3.32 (0.07)	0.31	0.754
	nose height	2.03 (0.05)	2.02 (0.04)	−0.70	0.485
	profile face length	10.67 (0.014)	10.68 (0.16)	0.68	0.494
	mouth width	5.62 (0.27)	5.65 (0.23)	1.14	0.255

Note: * indicates a statistically significant difference

Second, we used the total FF value of the N95 respirators as the dependent variable and considered face length, face width, chin length, chin width, nose length, nose width, nose height, profile face length, and mouth width as the independent variables. The step-up regression method was adopted. The test level α was selected as 0.1 when the independent variables were included and 0.15 when the independent variables were excluded. The possible factors influencing the total FF value of the N95 respirators were screened, and a multiple linear regression equation between the total FF value and the facial dimensions of the N95 respirators was established. Standardised partial regression coefficients were calculated to clarify the correlation between the independent and dependent variables in the equation. The analysis showed that face width (standardised estimate = 0.14) and nose length (standardised estimate = 0.20) both had a positive effect on the total FF value of the 3M 9132 respirator, and the influence of nose length was slightly greater than that of face width. Face width (standardised estimate = −0.14) had a negative effect on the total FF value of the NTPN95N respirator. Chin length (standardised estimate = 0.25) had a positive effect on the total FF value of the NTPN95N respirator, and chin length had a slightly greater effect than face width. Face width (standardised estimate = 0.11) and nose length (standardised estimate = 0.17) both had a positive effect on the total FF value of the WN-N95 fold style respirator, and the degree of influence of nose length was slightly larger than that of face width. Face width (standardised estimate = 0.11) and nose length (standardised estimate = 0.15) both had a positive effect on the total FF value of the WN-N95 cup style respirator, and the influence of nose length was slightly greater than that of face width. No facial dimensions with a statistically significant effect on the total FF value of the SW D918 respirator were identified. Table 5 presents the results of the multiple regression analysis for the total FF value and facial dimension characteristics of the five types of N95 respirators.

**Table 5:** Multi-factor analysis of total FF and facial dimensions

**N95 respirators**	**Variable**	**DF**	**Parameter** **Estimate**	**Standard** **Error**	**t Value**	**Pr > |t|**	**Standardized** **Estimate**
3M9132	Intercept	1	−2198.96	708.77	−3.1	0.002	0
	face width	1	149.11	63.23	2.36	0.019	0.14
	nose length	1	174.88	50.14	3.49	0.001	0.20

SW D918	Intercept	1	162.17	3.60	44.99	<0.0001	0

NTPN95N	Intercept	1	934.39	703.38	1.33	0.185	0
	face width	1	−177.50	77.04	−2.3	0.022	−0.14
	chin length	1	223.90	54.20	4.13	<0.0001	0.25

WN-95 Folding	Intercept	1	−1949.93	807.52	−2.41	0.016	0
	face width	1	131.08	72.04	1.82	0.070	0.11
	nose length	1	167.46	57.13	2.93	0.004	0.17

WN-95 Cup	Intercept	1	−1835.91	812.67	−2.26	0.025	0
	face width	1	130.45	72.50	1.8	0.073	0.11
	nose length	1	145.71	57.49	2.53	0.012	0.15

Note: The total FF of the N95 respirator was taken as the dependent variable, and face length, face width, chin length, chin width, nose length, nose width, nose height, side face length and mouth width were taken as the independent variables. The test level α of the included independent variable is 0.1, and the test level α of the excluded independent variable is 0.15.

### Fit prediction model of N95 respirators based on facial images

#### Construction of fit prediction model of N95 respirators based on facial images

For each participant, a frontal facial image and the FF value of the five N95 respirators were obtained. The facial image and FF value formed the sample. These samples were used to establish a mapping model from the facial image to the FF value.

First, a 3D facial model was reconstructed from each facial image using the deep learning method. Based on the key points of the 3D facial model, similar transformation operations such as rotation, translation, and scaling were performed on the original facial image to ensure consistency between the transformed facial image and a standard reference face image. Subsequently, based on the FF value, the facial images were divided into several categories. The FF value ranged from 0 to 200; therefore, the facial images could be classified into 201 categories. The current sample collection comprised only 299 images, which was a relatively small number. Hence, we merged the categories into four categories (or groups)—namely [0, 49], [50, 99], [100, 150], and [151, 200], with each group containing approximately 50 FF values. These samples were then used to train a convolutional neural network for classification. Thus, a ResNet18 was established for facial images to FF value categories. During testing, the categories and probabilities of each FF value were predicted from the image, and the four expected categories were used to determine the predicted FF value. The same method was used to establish predictive models for each of the five types of N95 respirators. The N95 respirator with the highest FF value was recommended.

#### N95 respirator fit prediction process

First, a camera was used to capture the colour facial image of a participant, and input it into the 3D facial modelling unit. The 3D face modelling unit obtained a rectangular area of the face through face detection methods and then used a convolutional neural network-based method to reconstruct the corresponding 3D face model of the colour image. Second, the facial colour was normalised, and the depth map synthesis unit regularised the original facial image and synthesised a depth map based on the reconstructed 3D facial model. Third, the normalised colour face and depth images were inputted into the respirator’s fit prediction unit to obtain the FF value.

**Fig. 4:**
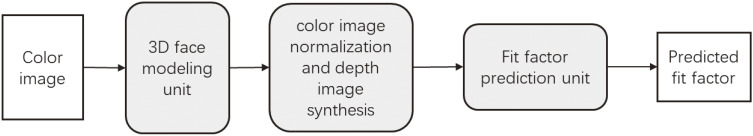
Diagram showing the N95 respirator fit prediction process

It is noteworthy that the fit prediction unit required offline training. To recommend different N95 respirators, it is necessary to establish corresponding fit prediction models for each type of N95 respirators and then use the N95 respirators model with the highest FF value as the recommendation result. Figure 4 shows the N95 respirator fit prediction process.

#### Evaluation of model prediction effect

A total of 54 medical workers participated in the model validation test. The fit prediction model was utilized to recommend suitable N95 respirators for the 54 medical staff members. These individuals then wore five different types of respirators for quantitative fit test, and the results are summarized in Table 6. The pass rates of the fit tests for N95 respirators were calculated for both the model-recommended group and the non-recommended group, and a statistical comparison was conducted to assess the difference between the two groups. The performance metrics of the fit prediction model—including accuracy, precision, recall, and F1 score—were calculated using the quantitative fit test results, with a FF ≥ 100 serving as the gold standard. Table 6 shows that the fit test pass rate in the model-recommended group (125/127 = 98.43%) was significantly higher than that in the non-recommended group (26/143 = 18.18%), with a chi-square value of 175.71 and P < 0.001, indicating a statistically significant difference.

$$\begin{array}{rl} & \text{Accuracy}=151/270*100\%=55.93\%\\ & \text{Precision}=125/127*100\%=98.43\%\\ & \text{Recall}=125/(125+117)*100\%=51.65\%\\ & \text{F1 score}=2*\text{Precision}*\text{Recall}/(\text{Precision}+\text{Recall})\\ & \;\;\;=0.68 \end{array}$$

**Table 6:** Evaluation of model prediction effect

**Model prediction**	**Quantitative fit test**		**Total**

	**Passed**	**Failed**	
Recommended	125	2	127
Not recommended	26	117	143

Total	151	119	270

## Discussion

This study revealed that the fit test results for the same individual wearing different types of N95 respirators were significantly different and that passing rates of the fit test for different types of N95 respirators were different. Facial dimensions of the wearers correlated with the fit test results of the N95 respirators. Table 4 and Table 5 shows analysis of the relationship between the total FF value of N95 respirators and the facial dimensions of participants. Cheng compared the relationship between five facial indicators and the FF value of half-surface filter respirators in 50 representative adults [13], and the conclusions were similar to those of this study. Studies conducted by Han et al. and Zhang et al. in North Korea and China, also showed that the fit of respiratory guards is related to the dimensional features of the wearers’ faces to a certain extent [14, 15].

Fit testing is considered the gold standard when using respirators and many countries have recommended legislative requirements for regular fit testing. The OSHA states that “The employer shall provide a medical evaluation to determine the employee’s ability to use a respirator, before the employee is fit-tested or required to use the respirator in the workplace” [6, 16–18]. The European Centre for Disease Prevention and Control states that filtering facepiece respirators require a fit test to ensure proper protection [19]. Similarly, the Health and Safety Executive in the UK states that fit testing should be performed to ensure that the respirator can protect the wearers [20].

The conventional N95 respirator fit quantitative test relies primarily on a respirator fit test. The high price of this medical instrument, the cumbersome testing process, and the need for wearers to cooperate for a long time make this method difficult to promote. Rapid testing of N95 fit has become an urgent problem. In this study, a 3D face model was reconstructed using computer vision technology, based on a colour image of the wearers’ faces. Subsequently, the colour face image was normalised according to the 3D face model, and a depth map was synthesised. Finally, a deep learning method was used to quickly predict the mask fit from the colour and depth images. The current state of the fit prediction model established in this study is a computer program that can be run by manual access to computer language commands and needs to run in the ubuntu18.04 system. In the foreseeable future, it will be transformed into a simpler Windows-based working program and even made into an instrument similar to a scanner so that it will greatly improve the convenience of N95 respirator fit test and reduce the cost of N95 respirator fit test.

Rapid selection of appropriate N95 respirators by human face image recognition can reduce the time cost of medical staff in selecting N95 respirators and the waste caused by choosing an N95 respirator with poor fit. This can reduce the risk of occupational exposure caused by the use of a poorly suitable N95 respirator and reduce the loss of medical personnel and treatment costs caused by occupational infection. If the research results on the distribution of facial dimension characteristics of the population and the fit test of the N95 respirators shared with the N95 respirator manufacturer, it can promote the N95 respirator manufacturer to adjust the production model of the N95 respirators according to the proportion of the population with different facial characteristics. If the model that can predict the fit of the N95 respirators according to the facial dimension characteristics of the population is developed into computer software or a machine that can run independently, it can be provided to various medical institutions for use. Medical workers may select N95 respirators with the highest FF value using prediction software based on the evaluation results.

Characteristics and limitations of the established model in this study: (1) The N95 respirator fit prediction model developed in this study was constructed and trained using participants’ facial dimension data and quantitative fit test results of respiratory protective devices. Different racial and ethnic groups exhibit distinct facial morphological characteristics, and incorporating diverse racial/ethnic populations into model training may enhance the model’s predictive performance. Owing to the constraints of the current research, the dataset was drawn entirely from medical staff in Beijing and multi-center or multi-ethnic validation would be a valuable direction for future research. (2) All N95 respirators should conform to applicable national or regional standards. Including respirators from various manufacturers may improve the model’s predictive accuracy. In this study, only five N95 mask models were included, with limited product specifications, which may not adequately represent the wide range of brands and models available for practical use. This limitation will be addressed in future research. (3) In this study, only objective data such as facial images and FF value were collected without considering subjective factors like wearer comfort or odor perception which can influence their choice. Future investigations will encompass participants’ subjective experiences wearing N95 respirators as reference factors for selection alongside incorporating them into the model. (4) The current fit prediction model requires operation within a Linux system environment by trained personnel. To enhance accessibility, the project team is working towards developing an independent program compatible with Apple, iOS, and Windows systems, enabling installation on mobile devices.

The appropriate selection of respirators is crucial for the occupational safety of medical personnel, as reflected in the specific requirements of the fit prediction model. The respirators recommended by the model should closely align with established standards; therefore, the accuracy of the fit prediction model is of paramount importance. In this study, the fit prediction model achieved an accuracy of 98.43%, demonstrating significant practical application value. Since selecting an appropriate respirator is a task that individuals perform infrequently, the one-time computational cost of the fit prediction process is less critical than achieving the highest possible level of accuracy. In future research, we intend to broaden the scope of this study by incorporating a more diverse sample of participants representing various racial and ethnic backgrounds, as well as including a wider range of N95 respirators from different brands, models, and countries of origin through a multicentred investigation. The current model based on ResNet18 is appropriate. In future research, more advanced architectures—such as ResNet50 or Efficient Net—would be explored in future studies for potential performance enhancement.

## Conclusion

By combining the human face image recognition obtained through computer vision technology with the fit test results of people with different facial dimensions who wore N95 respirators, a deep neural network model was adopted to establish the relationship between facial images and the FF of N95 respirators. N95 respirators meeting the FF value requirements could be quickly selected through facial image scanning. The N95 respirator with the highest FF value can be swiftly recommended to medical personnel based on facial dimensions in a non-contact manner to improve the wearing efficiency of the N95 respirator, and improve the prevention and control of respiratory transmitted diseases.

## Declarations

### Availability of data and material

The data and material of the current study are available from the corresponding author on reasonable request.
