# Correction to: Establishment of a fit prediction model of N95 respirator based on facial images

**DOI:** 10.1265/ehpm.26-00210

**Published:** 2026-09-26

**Authors:** Guifang Wang, Changwei Luo, Can Cui, Shengjin Wang, Jing Huang

**Affiliations:** 1 Department of Infection Control, Beijing You’an Hospital, Capital Medical University, No. 8, Xitoutiao, You’anmen Wai, Fengtai District, Beijing, China, 100069; 2 Department of Electronic Engineering, Tsinghua University, No. 30, Shuangqing Road, Qinghuayuan Street, Haidian District, Beijing, China, 100084; 3 Academy of Military Sciences, No. 1, Xianghongqi Dongmenwai, Haidian District, Beijing, China, 100091; 4 Department of Infection Control, Peking Union Medical College Hospital, Chinese Academy of Medical Sciences, Peking Union Medical College Hospital, No.1 Shuaifuyuan Wangfujing Dongcheng District, Beijing, China, 100730

**Correction to: *Environ Health Prev Med* 30, 101 (2025)**

**https://doi.org/10.1265/ehpm.25-00258**

After this article was published [1], it was discovered that there are calculation errors in the accuracy, recall and F1-score. and we will correct it as follows.

On page 7, in the Evaluation of model prediction effect section,
“Accuracy = 151/270 ∗ 100% = 55.93%
Precision = 125/127 ∗ 100% = 98.43%
Recall = 125/(125 + 117) ∗ 100% = 51.65%
F1 score = 2 ∗ Precision ∗ Recall/(Precision + Recall) = 0.68”
corrected to
“Accuracy = (125 + 117)/270 ∗ 100% = 52.59%
Precision = 125/127 ∗ 100% = 98.43%
Recall = 125/151 ∗ 100% = 82.78%”
F1score = 2 ∗ Precision ∗ Recall/(Precision + Recall) = 0.90”

The correction do not alter the study’s conclusions. Although the value of accuracy is slightly reduced, recall and F1-score are substantially improved after correction. The corrected calculations for accuracy, recall, and F1-score do not alter the overall research findings and academic arguments of this study; instead, they demonstrate that the predictive model achieves better overall performance. All main conclusions of this study remain valid, as supported by the updated quantitative results above.
